# Experiments on the Dynamic Behavior of Curved Glass Panes Subjected to Low-Velocity Impact

**DOI:** 10.3390/ma16237335

**Published:** 2023-11-25

**Authors:** Marcin Kozłowski, Kinga Zemła

**Affiliations:** Department of Structural Engineering, Silesian University of Technology, Akademicka 5, 44-100 Gliwice, Poland; kinga.zemla@polsl.pl

**Keywords:** curved glass, impact loading, dynamic, experiments

## Abstract

Curved glass enables designers to achieve unparalleled innovation in creating modern and undulating shapes for building enclosures. However, the curvature of panes changes the static and especially the dynamic behavior of the panes under loading. Studies on low-velocity impacts on curved glass have been limited and have primarily involved numerical studies. This paper experimentally investigates the dynamic response of cylindrically curved glass panes under a low-velocity impact. A flat, 5 mm thick, single-pane geometry with three curvature radii and the lack or presence of movement restraint is considered. Special attention is also paid to the variations caused by impacting bodies involving different stiffness, mass, and geometry parameters. It was found that flat plates have a lower capacity to dampen oscillations, resulting in longer decay times compared to curved panes. For impactors with a lower stiffness, the glass panes experience uneven oscillations at the moment of impact, followed by a chaotic period of transient vibrations before reaching a stationary state. This contrasts bodies with greater deformability in which the main dynamic behavior follows a more predictable pattern.

## 1. Introduction

Glass has been a popular material for building use for many decades. Its transparency has long been a key feature that has inspired designers to find innovative ways to incorporate it into architectural designs [1]. Today, glass is being used more and more frequently in construction, even as a load-bearing element in the form of glass roofs, facades, and balustrades, as well as glass beams and stairs [2]. The reason behind this, in addition to esthetic features, is the growing trend of maximizing natural sunlight in buildings, which has a positive impact on people who live and work indoors [3].

The rise of free-form architectural structures has led to the development and advancement of curved glass as a component of geometrically intricate building facades [4]. In this way, utilizing curved glass enables designers to achieve unparalleled innovation in creating modern and undulating shapes for building enclosures.

There is a significant difference in the structural behavior of flat and curved sheets as demonstrated by Pini et al. [5]. The curvature particularly stiffens spatially thin sheets and changes their static and especially dynamic behavior [6]. In the case of Insulating Glass Units (IGUs), the curvature of the panes can result in unexpected structural responses to static and climatic loads due to their increased spatial stiffness [7,8].

An aspect that is crucial and often underestimated in the design process is dynamic loads. These actions usually govern the final thickness of the glass and should not be overlooked. Even after physical contact between an impactor and a glass element has ended, the glass may continue to react dynamically for a significant period [9]. As a result, the glass may experience critical stresses much later than the initial impact [10]. This is especially apparent in glass elements with intricate shapes or in slender glass panes with openings in which the maximum stresses may occur during the later stages of a dynamic event [11].

Although curved elements have been extensively utilized in various applications, there has been limited research on low-velocity impact on shells. Ramkumar and Thaker [12] utilized Donnell’s approximations for thin shells and the Fourier series method to predict the transient response of a curved, laminated plate under low-velocity transverse impacts by a rigid object. Similarly, Christoforou and Swanson [13] employed the Fourier series method to derive a closed-form solution for the problem of simply supported orthotropic cylindrical shells. Lin and Lee [14] conducted experimental and numerical studies on the impact damage of laminated plates and cylindrical panels, finding that shell structures are more vulnerable to damage than plates when exposed to the same impact velocity conditions. The study by Palazotto et al. [15] shed more light on the utilization of nonlinear shell theory to obtain the impact response of cylindrical panels, predicting deflections and stresses by experimental means. Kim et al.’s results contribute to our understanding of the dynamic behavior of curved laminated composite structures [16]. Numerical computations were carried out to show the effects of curvature and stacking sequence on the impact response of cylindrical composite shells. It was found that the curvature significantly affects the dynamic behavior; in particular, the contact force exerted on a cylindrical composite shell increases with the curvature.

The topic of the dynamic response of curved shells to low-velocity impact has indeed been addressed in the literature; however, it pertained to other materials such as steel, composites, and various honeycomb structures [17,18]. Glass is an entirely different material with different Young’s modulus and damping properties, which influence its specific dynamic response. There are likely no strictly experimental research examples in the literature. It is probably caused by the fact that performing experimental studies is costly and challenging due to the fragility and smooth surface of the glass, which significantly limits the possibilities for the use of experimental equipment [19]. The novelty of the research results published in this article probably pertains to some of the initial studies of such structures made of glass. Another novelty lies in the analysis of various types of impactors involving different stiffness, mass, and geometry parameters.

In the case of glass, studies on low-velocity impact loads on curved glass have been limited and primarily involved performing numerical studies. Galuppi and Royer-Carfagni [20] analytically examined the effect of curvature on the shear coupling of glass plies through the interlayer using the traditional approach developed by Newmark. It was found that the response of a curved structure is greatly influenced by the axial force it undergoes, and such internal action is mainly governed, for fixed applied loads, by the boundary conditions at the extremities.

Sukhanova et al. performed a numerical analysis of the dynamic state of shallow shell laminated glasses [21]. Their work aimed to investigate the dependence of laminated glass’s dynamic deformation on the glass curvature. The laminated glass model with a dimension of 305 × 305 mm^2^ consisted of two glass panes with a thickness of 5 mm, laminated together with a PVB interlayer with a thickness of 1.52 mm. The curved samples were subjected to impact through contact with the 83 mm diameter smooth solid steel ball (2.3 kg). The results showed that the distribution of the maximum magnitude of the displacement vector and intensity strain could be traced with an increasing curvature parameter. The authors concluded that the maximum magnitude of the displacement vector decreases with increasing curvature parameters, but with a high curvature parameter, it can increase slightly. Moreover, the intensity stress increases with increasing curvature parameter until about 45 mm and then decreases. Sukhanova and Larin [19] studied the numerical dynamics of laminated glass panes with different curvatures (a curvature parameter ranging from 0 mm to 250 mm). Their work studied the influence of the curvature parameter on the frequencies and modes of composite panes involving the propagation of elastic waves in the linear state. It was found that, with a threshold value of the curvature parameter of 48.88 mm, the first natural frequency increased by more than 330% and then decreased.

In the current paper, the authors empirically investigate the dynamic response of cylindrically curved glass panes under low-velocity impact. A 5 mm thick, single-pane geometry with different radii of curvature and the lack or presence of movement restraint is considered. Special attention is also paid to the variations caused by an impactor involving different stiffness, mass, and geometry parameters.

## 2. Materials and Methods

### 2.1. Test Specimens

Plates with dimensions of 1000 × 1000 mm^2^ and a thickness of 5 mm, were made of soda-lime-silica glass. The length of the arc determined the dimension of the curved sheet. For all samples, fully tempered (toughened) glass was used. Before the heat treatment process, the edges were ground and polished.

Four glass plates were investigated: one was flat, while the other three had varying bending radii: 2821 mm, 1444 mm, and 1000 mm, which corresponds to an arch height of approx. 29, 57, and 86 mm, respectively. The minimum radius (1000 mm) was determined by production limitations for the glass thickness of 5 mm, while the others resulted from the division of the arch height of 86 mm (for the curved sample with a 1000 mm bending radius) into three parts.

### 2.2. Impacting Bodies

**Figure 1:**
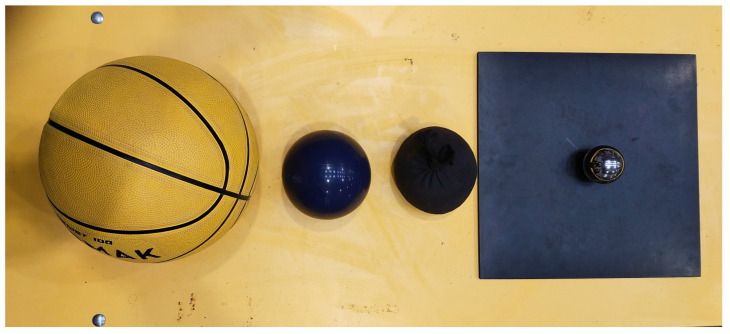
Impact bodies used in the study from the left: basketball ball, sand-filled rubber ball, fabric bag filled with peas (beanbag), steel ball on a rubber pad.

For the tests, impacting bodies with varying characteristics were selected, which involved different stiffness, mass, and geometry parameters, see Figure 1. A practically undeformable steel ball, a basketball (with a pressure equal to 0.62 bar), a rubber ball filled with sand, and a fabric bag filled with peas (beanbag) were used. A 7 mm thick rubber pad with dimensions of 300 × 300 mm^2^ was used with the steel ball to ensure safety and that the pane would not be damaged during the tests. It should be noted that this setup resulted in a semi-hard-body impact.

The parameters of the impacting bodies are summarized in Table 1.

### 2.3. Experimental Setup

**Figure 2:**
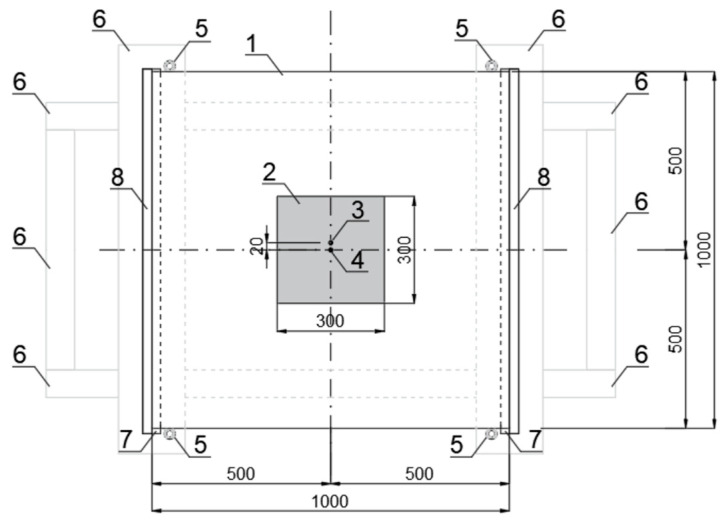
Top view of the test stand scheme: (1) glass plate, (2) rubber pad (used for steel ball impact), (3) bottom accelerometer, (4) inductive displacement sensor mounted from the bottom, (5) Teflon tubes that block movement in the direction parallel to the edge of the supports, (6) wooden frame, (7) Teflon strips on which the pane rests, and (8) additional Teflon strips (used in the variant with locked horizontal sliding).

**Figure 3:**
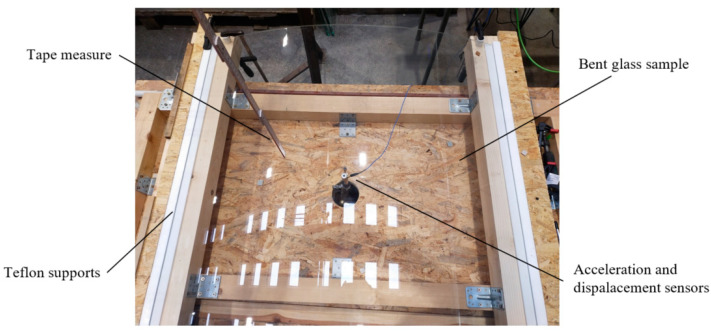
Test stand (top view).

**Figure 4:**
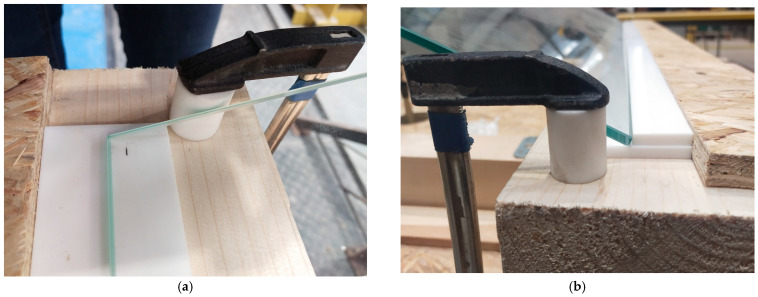
Detailed support arrangement of the test stand: (**a**) simple support; (**b**) restrained movement.

The test stand is presented in Figure 2 and Figure 3. It consists of four wooden beams on which Teflon flat bars, glass panes, and steel racks were placed to form a framework to attach the measuring tools that determine the height of the body drop. Figure 4 illustrates the plate locking elements required for two types of tests: simply supported slabs and those with restrained sliding (outside) perpendicular to the line of supports. Additional Teflon strips were used for wedging the plates. In addition, Teflon tubes were fitted in the corner zones of the sample to prevent the plates from moving horizontally in parallel directions to the support lines (as shown in Figure 4). It should be noted that the horizontal restraints only worked for the curved panes, while, for the flat panels, they were inactive. This occurred because, during impact, the curved sample flattens; its straight edges move to the outsides (the sample opens up), and horizontal reactions occur. However, the flat sample behaves differently during the impact; it deflects in the opposite direction of the impact, and its edges move closer to the center of the sample (chord shortening). Thus, restraining the movement of the flat sample was not effective, as it did not have a horizontal reaction on the supports during impact.

The sensors were attached to the pane and then connected to two measurement devices: Alitec™ QACQ, Alitec, Łódź, Poland [22] and QuantumX - MX840B, HBM, Poznań, Poland [23]. The first recorded the acceleration of a selected point on the surface using an accelerometer weighing 10.5 g, with a maximum measuring range of ±4900 m/s^2^ and a frequency range of 1 to 5000 Hz ± 5%. This type of accelerometer was chosen due to its relatively small size and low mass to minimize its influence on the dynamic behavior of thin plates and the measured values. The latter was a Linear Variable Differential Transformer (LVDT) to collect displacement data.

**Figure 5:**
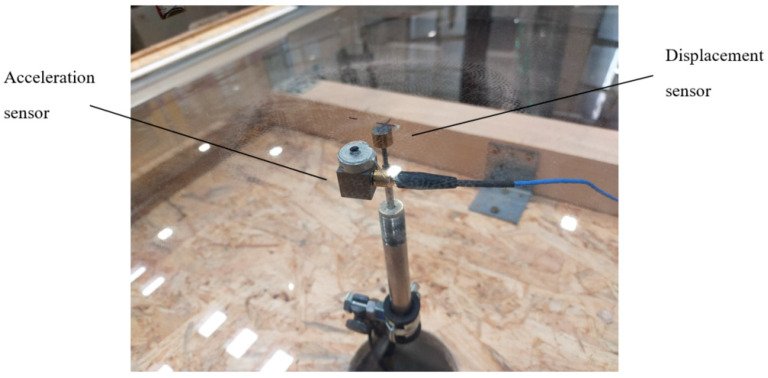
Sensors attached to the sample.

To carry out this research, two highly specialized measuring devices were necessary. The Catman^®^ 5.6.1 [24] software was used to collect displacement sensor data, while VIDIA^®^ [25] was used to gather data from a QACQ device (acceleration). The measurement systems used in this research allowed for the independent recording of results. The QACQ device was able to sample at a frequency of 64,000 Hz, while the HBM QuantumX device was able to sample at 19,200 Hz. Figure 5 presents the sensors that were attached to the pane—the displacement sensor was attached at the center point of the pane, while the acceleration sensor was shifted 20 mm in the direction of the unsupported edge from the center point. Both the LVDT and the accelerometer were securely placed on the bottom surface of the glass sample. Details of the experimental campaign can be found in a master’s degree thesis devoted to the dynamical response of the curved glass pane subjected to impact [26].

### 2.4. Experimental Methodology

Four types of experiments were carried out using the impact bodies mentioned in Section 2.2 to investigate the dynamic behavior of curved glass panes under low-velocity impact. Each body was dropped from a different height, ensuring it impacted the panes with the same kinetic energy of 2.5 J according to EAD 210005-00-0505 [27]. The calculations were based on the potential energy equation, which indicates that a heavier body must fall from a lower height to achieve the same energy as a lighter body. The drop heights derived analytically for the individual bodies are presented in Table 2. However, it should be noted that achieving this precision during experiments was practically impossible, and this could affect the measured values and their scatter.

The glass panes were directly impacted at the center point. The objects were directly dropped on the glass pane, except the steel ball, which had a rubber pad of dimensions 300 × 300 mm^2^ and thickness of 7 mm placed on it. The initial stage involved analyzing a flat reference sheet by subjecting it to six impacts with each object. Subsequently, curved sheets were tested in two variations. The first variation involved restraining the horizontal movement of the sample with Teflon flat bars, while the second was a simply supported scheme without an additional restraint (see Figure 4). Similar to the testing of the reference samples, curved glasses were subjected to six impacts. Regular checking of the displacement sensor was critical during the experiments as the vibrations from the impact could fall off the sensor from the tested samples. It is worth emphasizing that each of the impactors, except for a fabric bag filled with peas (beanbag), was caught after the first drop (after the first impact on the glass pane) to avoid a second impact. After dropping on the sample, only the beanbag was allowed to remain on it until the measurements were completed. Moreover, in each attempt, the beanbag hit the sample with a different shape due to its irregular structure. The above-mentioned facts could affect the final results due to the increased weight of the element.

## 3. Results and Discussion

To comprehensively study how glass panes react to low-velocity impact, the experimental results were analyzed by examining the displacement history of the glass panes, the decay time, and the maximum accelerations over time.

### 3.1. Displacement Response

**Figure 6:**
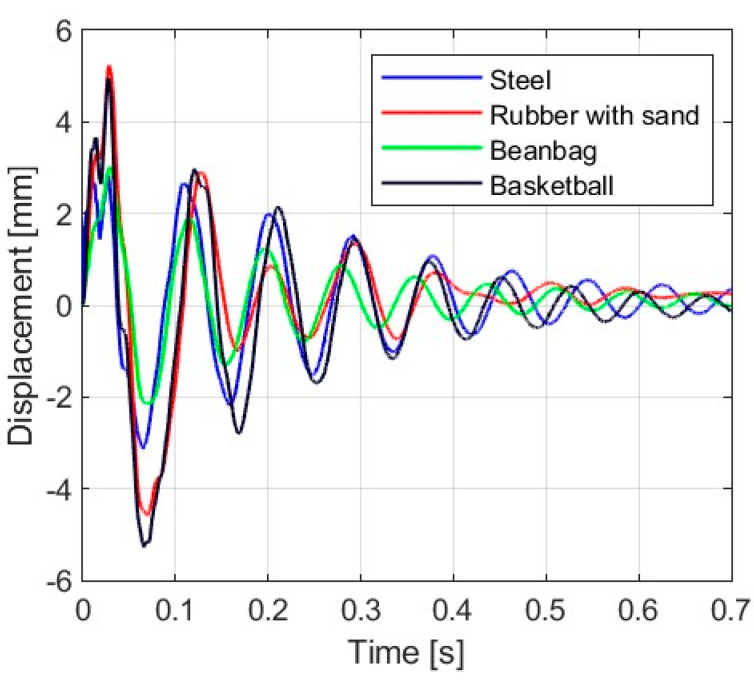
Results of experiments for the flat glass pane (simply supported).

**Figure 7:**
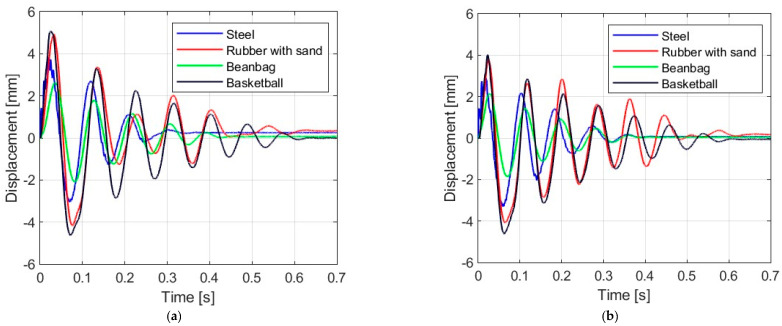
Results of experiments for the glass pane with radius R = 2821 mm: (**a**) simply supported, (**b**) restrained movement.

**Figure 8:**
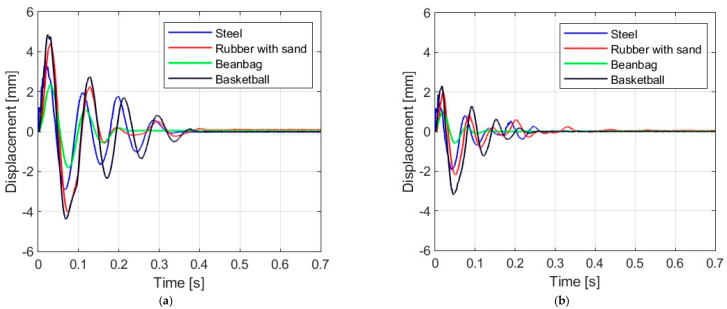
Results of experiments for the glass pane with radius R = 1444 mm: (**a**) simply supported, (**b**) restrained movement.

**Figure 9:**
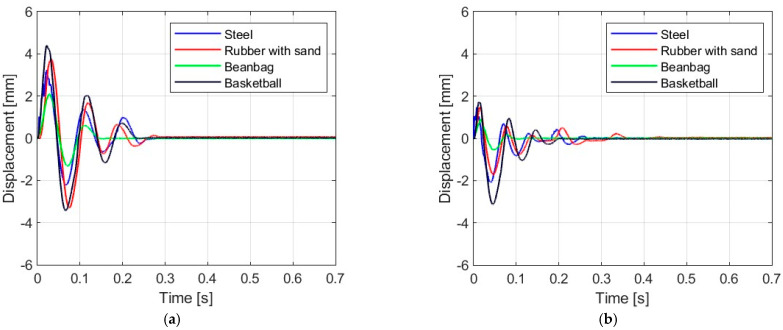
Results of experiments for the glass pane with radius R = 1000 mm: (**a**) simply supported, (**b**) restrained movement.

**Figure 10:**
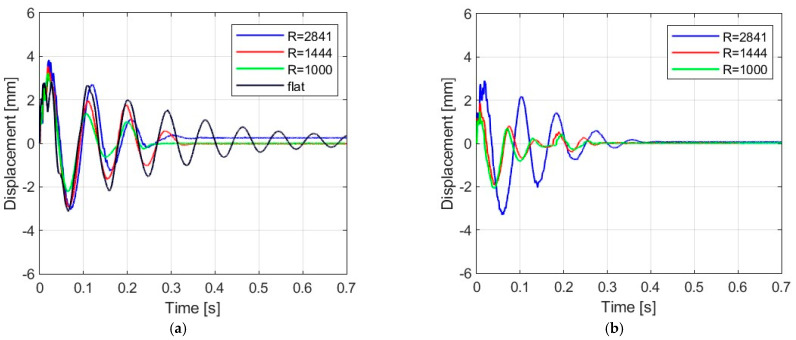
Results of experiments with steel ball: (**a**) simply supported, (**b**) restrained movement.

**Figure 11:**
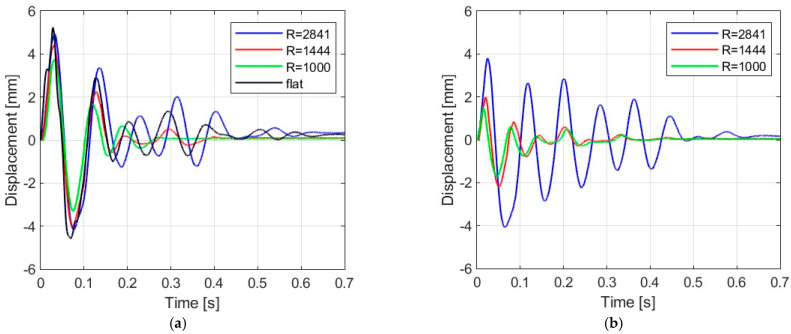
Results of experiments with rubber ball filled with sand: (**a**) simply supported, (**b**) restrained movement.

**Figure 12:**
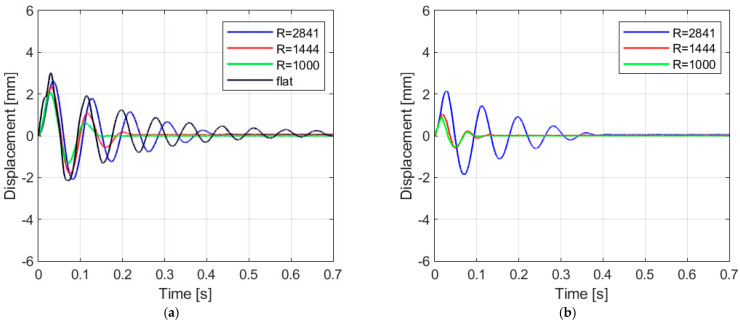
Results of experiments with fabric bag filled with peas: (**a**) simply supported, (**b**) restrained movement.

**Figure 13:**
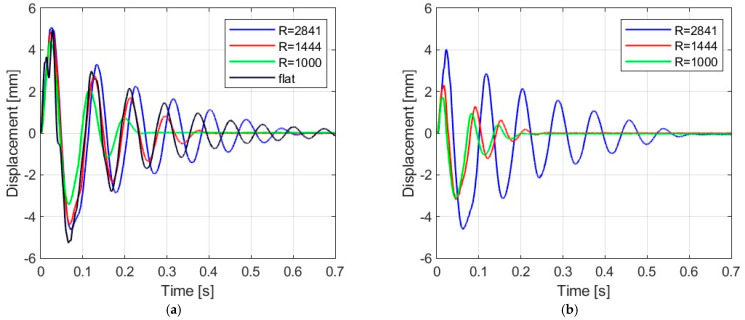
Results of experiments with basketball: (**a**) simply supported, (**b**) restrained movement.

Figure 6, Figure 7, Figure 8 and Figure 9 show the displacement histories measured in the center of the glass panes during the tests for all impact bodies for flat and curved panes. The figures also show the difference in the dynamic behavior under two boundary conditions: simply supported and restrained movement. To maintain clarity, only one randomly chosen analysis result within each series is shown. To gain a deeper understanding of the subject of this research, the histories of the displacement for the centers of the panes have also been compiled separately for each impactor, considering different bending radii, see Figure 10, Figure 11, Figure 12 and Figure 13. Table 3 presents detailed data in the form of tables regarding the mean values with coefficients of variation.

Comparing Figure 6, Figure 7a, Figure 8a, Figure 9a and Figure 10a showing the results for simply supported conditions, it can be noticed that the impactor significantly affects the dynamic behavior of the glass panes, and several observations can be made. The results indicate that the flat sheet has a lower capacity for energy dissipation, and its oscillation amplitudes decline exponentially with time. This is in contrast to the curved sheets, which experienced fewer oscillations. The curved plate (2821 mm) also exhibited a similar pattern of decreasing vibration amplitude in time for both support conditions. However, the damping for the curved plates (1444 mm and 1000 mm) was more erratic, with vibration amplitudes not decreasing exponentially. The damping waveforms for the plates were very similar, but there was a noticeable difference in the value of the first peak displacement. A common observation in all plots was the presence of noise in the first displacement peak, which was most likely due to the ball hitting the rubber pad first. Although a 2821 mm curved plate had a greater maximum displacement when simply supported, its damping time was comparable to that of a restrained pane. The ability to dissipate energy decreased as the bending radius increased. This ability was further demonstrated when comparing a 2821 mm slab with 1444 mm and 1000 mm panes. The results confirmed that 1444 mm and 1000 mm panes with restricted movement exhibited a similar course of displacements in time. The impact that resulted in a chaotic change in displacements over time was in the case of the rubber ball filled with sand. The trend of a flat sheet having a longer vibration damping time remained consistent. Similar to the damping course for a steel ball, the damping courses for sheets with a bending radius of 1444 mm and 1000 mm were comparable. For the beanbag, it was observed that the damping of the 1444 mm and 1000 mm panes were very similar. However, comparing them to the 2821 mm surface, it can be concluded that energy dissipation occurred much faster in more-curved sheets. Similarly, comparing panes with the same bending radii but different support methods, it was found that panes with locked travel tended to dampen vibrations quicker. The flat plate and the 2821 mm curved pane exhibited an exponential decrease in vibration amplitude. For the fabric bag filled with peas (beanbag), the values of minimal displacement were significantly smaller than the maximal (positive) ones. This can be caused by an impactor that remained on the sample after the impact. For restrained movement support, the minimal (negative) value of displacement was greater than the positive values (except for beanbag impact).

### 3.2. Decay Time

**Figure 14:**
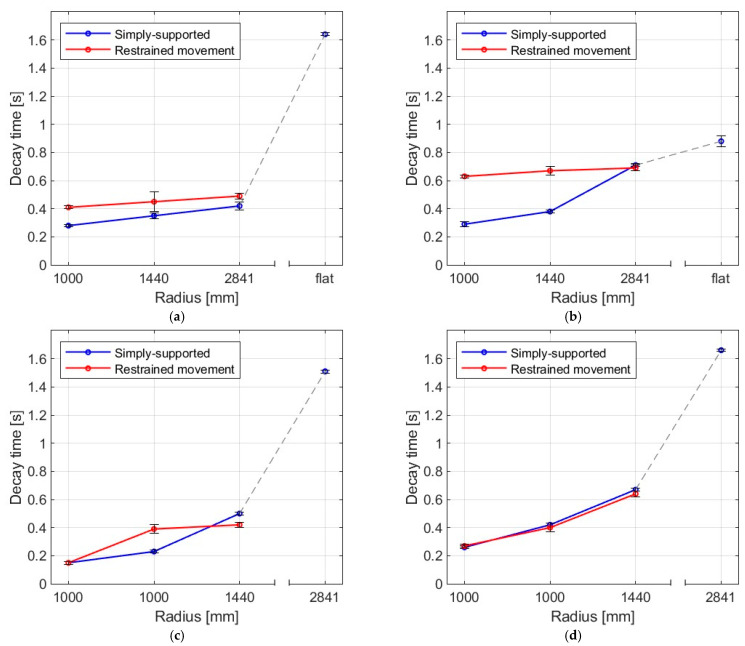
Mean decay time of the plates as a result of impact by (**a**) steel ball, (**b**) rubber ball filled with sand, (**c**) fabric bag filled with peas, (**d**) basketball.

**Figure 15:**
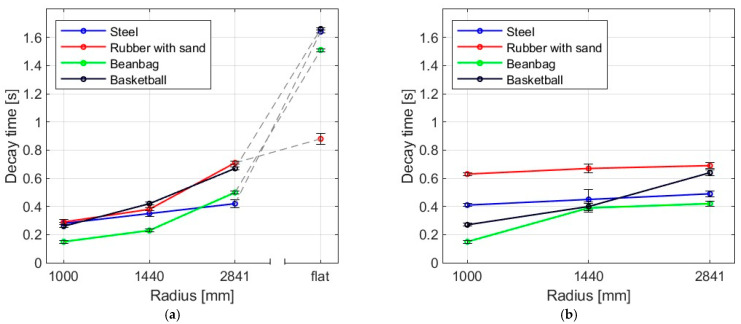
Mean values of decay time for four impactors in (**a**) simply supported, (**b**) restrained movement supports.

Figure 14 and Figure 15 show the decay times for all impact bodies for the flat and curved panes. The figures also show the differences between two boundary conditions: simply supported and restrained movement. Table 4 presents detailed data in the form of a table regarding the mean values and coefficients of variation.

It was observed that the larger the bending radius, the lower the energy dissipation ability and the longer the decay time. For flat panes in a simply supported arrangement, this effect was significant. For the simply supported arrangement, it can be noted that the values for the fabric bag filled with peas (beanbag) for smaller radii were the smallest of all, whereas, for greater radii (2821 and flat), they were greater than for the steel ball and the rubber ball with sand, respectively. This unexpected outcome could be explained by the fact that, for the impact series with a fabric bag with peas, the impactor was not removed after the first impact. This results in increasing the oscillatory period; thus, the decay time also increases.

### 3.3. Maximum Acceleration

**Figure 16:**
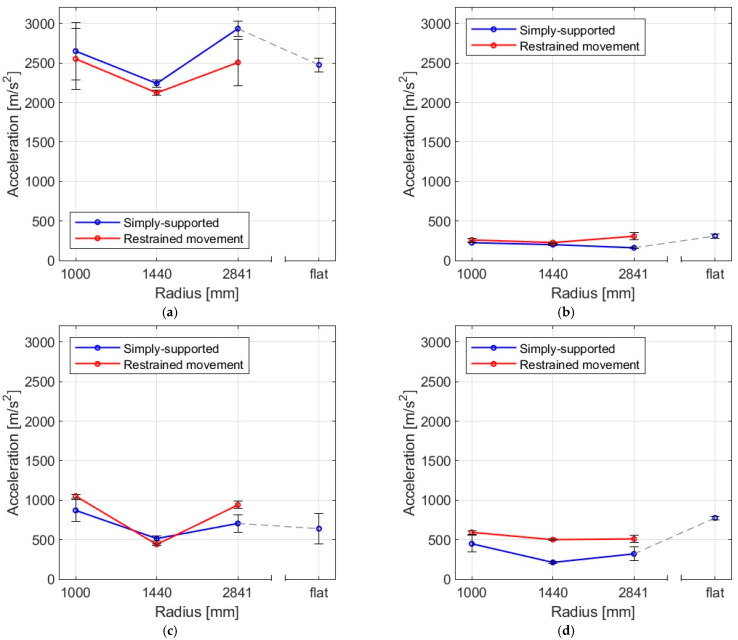
Mean acceleration of the panes as a result of impact by (**a**) steel ball, (**b**) rubber ball filled with sand, (**c**) elastic bag filled with peas, (**d**) basketball.

**Figure 17:**
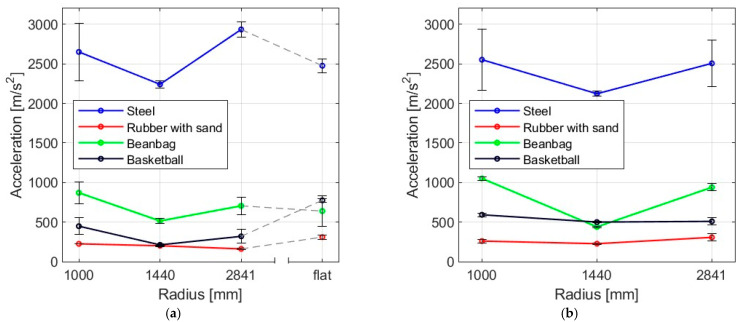
Mean values of acceleration for four impactors in (**a**) simply supported, (**b**) restrained movement supports.

The results of testing glass plates with varying bending radii, both freely supported and locked with sliding outside the arc and struck with four different bodies are presented in this section. Figure 16 and Figure 17 and Table 5 provide a summary of the average maximum acceleration values, including their standard deviation.

The highest values of mean acceleration of the sample were observed to be achieved by the steel ball impact, whereas the smallest values were obtained for a rubber ball with sand. Moreover, for most impactors, it can be seen that, for samples with a radius of 1444 mm, the accelerations were the lowest. After analyzing the data presented, it can be inferred that the steel ball resulted in the highest accelerations. On the other hand, the rubber ball filled with sand caused the least acceleration. However, the results do not offer a definite conclusion on how the bending radius of the slab and its support method affect the impulse load acceleration. Accelerations and similar quantities are highly prone to aliasing errors [28]. This is also reflected in the values of the coefficients of variation.

## 4. Conclusions and Further Work

This paper presents the experimental results of dynamic tests of curved glass panes under low-velocity impact with simply supported and restrained boundary conditions. It investigates the variations caused by an impactor involving different parameters of stiffness, mass, and geometry. In this study, a 5 mm thick pane with a geometry of 1000 × 1000 mm^2^ was examined.

From the experiments performed, the following conclusions were drawn.

Flat plates have a lower capacity to dampen oscillations, resulting in longer decay times compared to curved panes. Smaller bending radii in the panels lead to lower displacement values and faster dissipation of impact energy. This aligns with the observation that curved elements are generally more rigid than flat ones.

The size of the contact area plays a crucial role in determining the displacement. A smaller contact area concentrates the impact energy in a smaller point, resulting in greater displacement.

The geometry of the impactor also affects the contact area. The behavior of the glass varies depending on the type of impact body. For impactors with lower deformability, the glass panes experience uneven vibrations at the moment of impact, followed by a chaotic period of transient oscillations before reaching a stationary state. This is in contrast to bodies with greater deformability in which the main dynamic behavior shows a more predictable pattern.

## Figures and Tables

**Table 1:** Parameters of impact bodies.

Impactor	Mass of Impactor [g]	% of Glass Weight	Diameter [mm]
Steel ball	510.25	4.08	50
Basketball	483.35	3.87	220
Rubber ball filled with sand	891.24	7.13	107
Fabric bag filled with peas	506.24	4.05	104/120 ^1^

^1^ Measured in a hanging position/resting on a flat surface.

**Table 2:** Parameters of impacts.

Impactor	Mass [g]	Drop Height Derived Analytically [m]
Steel ball	510.25	0.499
Basketball	483.35	0.545
Rubber ball filled with sand	891.24	0.287
Fabric bag filled with peas	506.24	0.510

**Table 3:** Values of maximal and minimal displacements for impactors and four radii of the panes.

Impacting Body		Flat Pane	SS ^1^	RM ^2^	SS	RM	SS	RM
Steel ball	Max.	2.82 ± 0.02	3.85 ± 0.02	2.91 ± 0.01	3.52 ± 0.02	1.87 ± 0.01	3.25 ± 0.02	1.46 ± 0.02
	Min.	−3.09 ± 0.03	−3.03 ± 0.02	−3.32 ± 0.03	−2.89 ± 0.02	−1.93 ± 0.02	−2.26 ± 0.04	−2.07 ± 0.02
Rubber ball filled with sand	Max.	5.29 ± 0.05	4.93 ± 0.07	3.82 ± 0.03	4.34 ± 0.06	1.97 ± 0.02	3.74 ± 0.04	1.47 ± 0.02
	Min.	−4.62 ± 0.05	−4.31 ± 0.11	−4.10 ± 0.03	−3.93 ± 0.09	−2.13 ± 0.05	−3.21 ± 0.07	−1.67 ± 0.05
Fabric bag filled with peas	Max.	2.95 ± 0.06	2.21 ± 0.04	2.12 ± 0.01	2.33 ± 0.02	1.06 ± 0.02	2.07 ± 0.03	0.91 ± 0.01
	Min.	−2.10 ± 0.05	−2.07 ± 0.05	−1.83 ± 0.02	−1.78 ± 0.02	−0.61 ± 0.02	−1.31 ± 0.02	−0.55 ± 0.01
Basketball	Max.	4.97 ± 0.02	5.11 ± 0.05	4.04 ± 0.03	4.81 ± 0.06	2.29 ± 0.01	4.47 ± 0.12	1.73 ± 0.01
	Min.	−5.25 ± 0.02	−4.60 ± 0.04	−4.63 ± 0.02	−4.31 ± 0.05	−3.16 ± 0.02	−3.54 ± 0.12	−3.11 ± 0.01

^1^ SS stands for simply supported. ^2^ RM stands for restrained movement.

**Table 4:** Values of maximal and minimal displacements for different impactors and four radii of the panes.

Impacting Body	Flat Pane	R = 2821 mm		R = 1444 mm		R = 1000 mm	
		SS ^1^	RM ^2^	SS	RM	SS	RM
Steel ball	1.64 ± 0.01	0.42 ± 0.03	0.49 ± 0.02	0.35 ± 0.02	0.45 ± 0.07	0.28 ± 0.01	0.41 ± 0.01
Rubber ball filled with sand	0.88 ± 0.04	0.71 ± 0.01	0.69 ± 0.02	0.38 ± 0.01	0.67 ± 0.03	0.29 ± 0.02	0.63 ± 0.01
Fabric bag filled with peas	1.51 ± 0.01	0.50 ± 0.01	0.42 ± 0.01	0.23 ± 0.01	0.39 ± 0.03	0.15 ± 0.01	0.15 ± 0.01
Basketball	1.66 ± 0.01	0.67 ± 0.01	0.64 ± 0.01	0.42 ± 0.01	0.43 ± 0.03	0.26 ± 0.01	0.27 ± 0.01

^1^ SS stands for simply supported. ^2^ RM stands for restrained movement.

**Table 5:** Mean maximal values of the acceleration of the sample during tests.

Impacting Body	Flat Pane	R = 2821 mm		R = 1444 mm		R = 1000 mm	
		SS ^1^	RM ^2^	SS	RM	SS	RM
Steel ball	2476 ± 88	2934 ± 99	2506 ± 293	2241 ± 47	2122 ± 31	2650 ± 363	2552 ± 390
Rubber ball filled with sand	311 ± 30	162 ± 13	308 ± 47	202 ± 10	228 ± 8	226 ± 3	261 ± 22
Fabric bag filled with peas	640 ± 195	706 ± 111	940 ± 58	515 ± 33	440 ± 29	871 ± 141	1052 ± 157
Basketball	776 ± 24	321 ± 86	510 ± 122	213 ± 12	501 ± 41	449 ± 104	593 ± 75

^1^ SS stands for simply supported. ^2^ RM stands for restrained movement.

## Data Availability

Data is available on request.
